# The Curious Anomaly of Skewed Judgment Distributions and Systematic Error in the Wisdom of Crowds

**DOI:** 10.1371/journal.pone.0112386

**Published:** 2014-11-18

**Authors:** Ulrik W. Nash

**Affiliations:** Strategic Organization Design, University of Southern Denmark, Odense, Denmark; Cajal Institute, Consejo Superior de Investigaciones Científicas, Spain

## Abstract

Judgment distributions are often skewed and we know little about why. This paper explains the phenomenon of skewed judgment distributions by introducing the augmented quincunx (AQ) model of sequential and probabilistic cue categorization by neurons of judges. In the process of developing inferences about true values, when neurons categorize cues better than chance, and when the particular true value is extreme compared to what is typical and anchored upon, then populations of judges form skewed judgment distributions with high probability. Moreover, the collective error made by these people can be inferred from how skewed their judgment distributions are, and in what direction they tilt. This implies not just that judgment distributions are shaped by cues, but that judgment distributions are cues themselves for the wisdom of crowds. The AQ model also predicts that judgment variance correlates positively with collective error, thereby challenging what is commonly believed about how diversity and collective intelligence relate. Data from 3053 judgment surveys about US macroeconomic variables obtained from the Federal Reserve Bank of Philadelphia and the Wall Street Journal provide strong support, and implications are discussed with reference to three central ideas on collective intelligence, these being Galton's conjecture on the distribution of judgments, Muth's rational expectations hypothesis, and Page's diversity prediction theorem.

## Introduction

We measure and navigate our environment by making intuitive judgments, but these are fallible. By also giving weight to judgments made by others we can diminish our mistakes. The mean of many intuitive judgments, made by numerous different people, is accurate when judgments scatter around the truth. In fact, the mean is perfect when judgments scatter in symmetry around the truth, because then all mistakes of underestimation are matched by counterpart errors of overestimation. However, when the weight of judgments distribute in greater proportion on either side of the truth, the mean has error. Indeed, systematic error in the mean of judgments exists to the extent these distributions can be predicted. Given the trust bestowed upon popular judgment in democratic societies, it would have considerable implication for outcomes of decision making if such a phenomenon of predictability existed, because it would imply an avoidable type of mistake is currently being made in many domains, from the misdiagnosis of patients by consensus seeking doctors, to the misallocation of resources by consensus seeking managers, investors, and politicians. From that perspective this paper brings bad news, because it contains evidence of systematic error in the wisdom of crowds. However, there is also potentially good news, because collective error appears predictable by the way judgments observably scatter.

Judgment distributions are often curiously skewed, something long known [1] [2] [3], but something we have deferred efforts to understand. Here I argue that when people use cues to make inferences about their environment, when people use these cues with adeptness, and when the environment is extreme compared to the central tendency of peoples' prior experience, then skewed judgment distributions occur with high probability. Moreover, collective error can be inferred from the degree and direction of judgment distribution skew, and from the degree of judgment distribution variance, implying that judgment distributions are shaped by cues, and are cues themselves for the wisdom of crowds; decision makers can infer collective intelligence by the shape of judgment distributions, and can moderate their confidence in popular judgment accordingly. We can even hope to repair the systematic error of our collective intelligence, and gain greater knowledge about our world. One candidate procedure for doing that is outlined in the discussion.

I base my arguments on the augmented quincunx (AQ) model of sequential and probabilistic cue categorization by neurons of judges. The model is introduced shortly, and I conduct tests using 3053 distributions of judgments made by economists about the US economic system, published by the Federal Reserve Bank of Philadelphia, and the Wall Street Journal. The AQ model finds strong support, and I discuss what that means for three important ideas on collective intelligence, namely Galton's conjecture on the distribution of judgments, which Galton stated, unbeknownst to most, in his seminal paper on the wisdom of crowds [1], Muth's rational expectations hypothesis [4] [5], which became the foundation of modern economics, and Page's diversity prediction theorem [6], which is often cited to promote social diversity.

## The AQ Model of Judgment

Neurons that categorize and accumulate information about the environment have been modeled with success by researchers in order to understand the neural basis of choice [7] [8]. The cognitive mechanism studied involves competing neurons tuned to opposing hypotheses, whose firing rates indicate their level of confidence, and where these firing rates or levels of confidence are accumulated by other neurons positioned further downstream to generate an overall inference. When discrete packets of information consistent or inconsistent with competing hypotheses arrive at the brain, an accumulating variable is thereby created, which breaches one of two decision thresholds after a while. These models give researchers good reason to believe information clarity determines the speed at which people choose A over B (or B over A) and the amount of evidence needed to decide. But while current models thereby advance our understanding of binary choice, they cannot explain situations where people are required to form refined judgments.

So let us consider an alternative model of intuitive judgment, and let us, in the spirit of Galton's seminal study, assume judges are competitors trying to guess the weight of an ox at the West of England Fat Stock and Poultry Exhibition.

### The Problem of Discriminating

The problem faced by our judges involves discriminating between the weight of the particular ox presented to them, 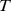, and the typical weight of oxen, 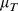, the latter being common knowledge gained through experience.

Judges cannot measure 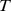 directly, but oxen have numerous perceptible regions, 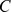, correlating with 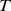. Judges use these regions to make inferences about how much the ox weighs. I follow standard practice and call these regions *cues*, while assuming they are stochastically independent. Regions might include, for instance, the height of the ox, or the degree to which its ribs are showing.

Across the population of all oxen, particular cues share the same correlation with weight, but they have various magnitudes, with some shoulders, say, being larger than others. The information value of the particular cue, 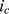, derives from both magnitude and correlation.

Typical cues have zero information value, while information values in general are distributed in symmetry around this level; particular cues contain more or less information by which to discriminate 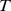 positively or negatively from 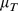. For example, while there is a typical degree of rib visibility, the visibility for any particular ox will be higher or lower, with more visible ribs informing judges they face an abnormally light ox 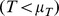.

In their attempt to discriminate 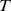 from 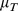, judges discriminate cues from what is typical, gathering evidence across all cues before guessing. I call this process *categorization*, because cues are either greater or smaller than their central tendency. Cues at the central tendency are referred to as *typical* cues.

### Categorizing Cues by Voting Neurons

**Figure 1:**
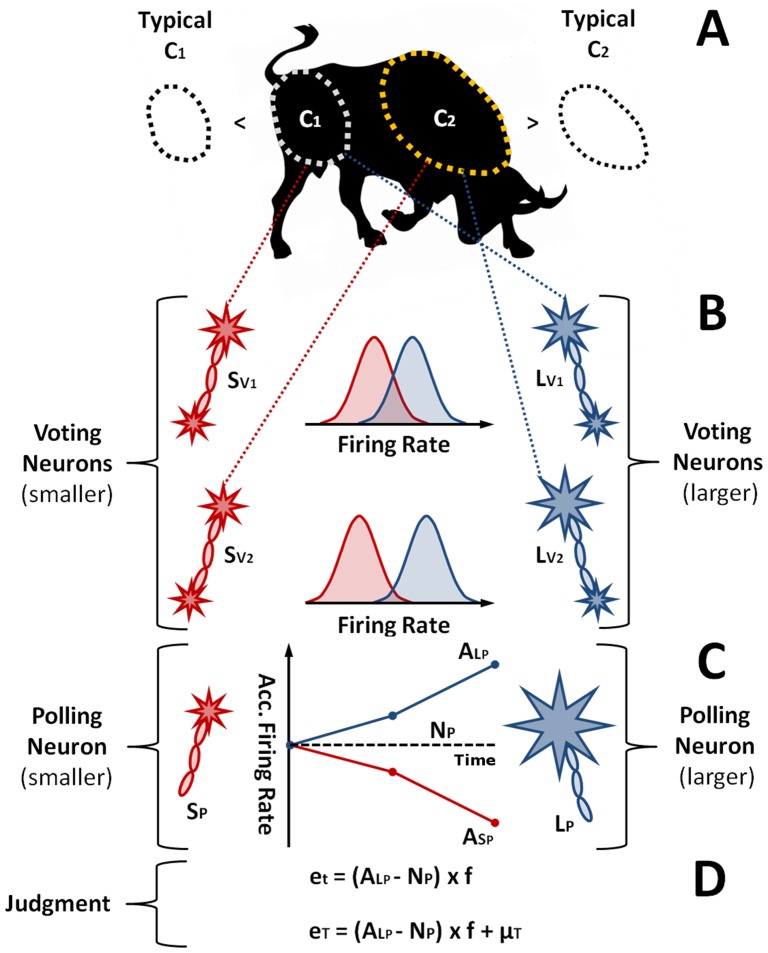
Information categorization by neurons leading to intuitive judgment. **A**. The weight of an ox must be judged. This true value cannot be observed directly, but numerous perceptible parts of the ox correlate with how much it weighs. These parts, here indicated as shoulder and rump, are cues (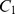 and 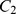), and their validity as indicators of weight are either larger or smaller than what is typical for such cues, with validity relative this reference point determining their information content. **B**. Information categorization by *voting* neurons. Two populations of voting neurons (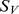 and 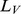) support opposing hypotheses about the true value, in the present case, that weight is larger or smaller than typical. Each population contains neurons tuned to particular cues, so that for each cue there are two competing neurons. As visualized here by the size of dendrites, these neurons moderate their firing rates according to how consistent information is with their supported hypothesis, with the neuron displaying greatest activity wining the right to encode information as consistent with its preference. This information categorization process is fallible because firing rates of voting neurons vary around their appropriate levels, as visualized here using probability distributions. **C**. Evidence accumulation by *polling* neurons. Polling neurons (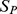 and 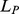) receive instructions from counterpart voting neurons to increase or decrease their firing rates above normal levels (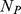). More specifically, defeated voting neurons send inhibitory instructions, while winning neurons send instructions encouraging greater activity. As streams of instructions are received, two accumulating variables are thereby established 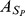 and 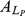. **D**. Intuitive judgment. The firing rate of the most active polling neuron, in this case 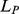, is ultimately converted to judgment by scaling this rate using an appropriate factor (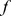). This provides an inference about the true value's degree of extremeness (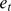), while subsequent addition of the typically encountered true value, 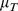, provides an inference about the true value in absolute terms (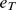).

The process of categorizing leads to refined judgment (Figure 1). Consistent with models of binary choice, refined judgment involves two neuron classes. The first class is represented by two populations, 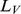 and 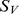, which contain neurons tuned to different regions of cues, and which respond with mean spike rates proportional to the information presented in their preferred region; these are *voting* neurons, as indicated by the chosen subscript, while 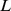 and 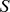 indicate their preference for competing hypotheses “larger than typical” and “smaller than typical”. The second class is represented by two counterpart neurons, 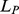 and 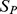, which receive and accumulate evidence from 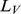 and 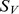 respectively; these are *polling* neurons. Once again the chosen subscript indicates neuron class, while 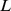 and 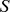 again indicate preferences for the hypotheses “larger than typical” and “smaller than typical”.

I assume that voting neurons remain unresponsive to cues outside their preferred region, implying that for every cue there are two active voting neurons, one in 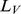 and one in 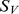. Voting neurons in 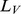 and 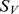 represent the competing hypotheses 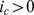 and 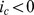 respectively, and each population reveals its level of confidence through the firing rates of active members. For higher values of 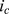, the mean spike rate across 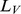, denoted 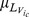, rises linearly, while the mean spike rate across 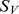, denoted 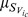, falls by the same absolute magnitude:
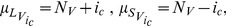
(1)where 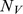 is the mean firing rate of the neuron activated by the typical cue.

Opposing neurons in 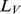 and 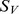 compete using their response to 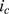, with the neuron demonstrating greatest activity winning the right to encode 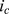 as being compatible with the hypothesis it supports. This evidence is subsequently passed to the counterpart polling neurons in 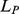 and 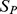 using the following instructions:
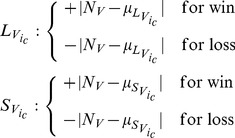
(2)


In words, when voting neurons win their competition, they instruct their counterpart polling neuron to increase its firing rate by an amount equal to the difference between the voting neuron's usual response, and the firing rate occurring under typical circumstances. Losing has the effect of reducing firing rates by the same magnitude.

### Noise and Categorization Error

If voting neurons could respond to 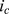 using only their mean firing rate, then battles between voting neurons would lead to perfect categorization because the spike rate within 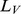 would always surpass the spike rate within 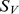 when 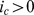, while the spike rate within 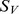 would always surpass the spike rate within 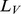 when 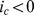. But firing rate variance introduces the possibility of categorization error.

Variance introduces the possibility that 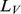 displays greater confidence even though 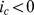, or that 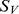 displays greater confidence even though 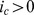. In each case 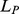 and 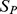 are given false instructions, implying that an error of categorization has occurred.

Although contemporary evidence suggests that neuronal responses are more accurately captured by the Poisson distribution [9], for the purpose of understanding categorization error, the Gaussian distribution has been used pragmatically since Thurstone's early ideas on comparative judgment [10]. Continuing this practice, the probability of categorization error can be understood by following two steps: First, we subtract the distribution of firing rates for the active neuron in 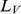, from the distribution of firing rates for the competing neuron in 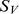, and second, we integrate the resultant Gaussian distribution from 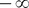 to 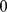. This yields:
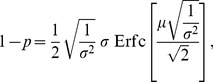
(3)where 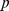 denotes the probability of correct categorization, 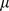 is the difference between the mean firing rates of active voting neurons, and 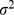 is the sum of their firing rate variance.

Differentiating error equation (3) with respect to 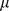 and 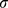 reveals that frequencies of categorization error increase with 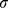, and decrease with 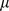; when voting neurons vary their firing rates more, or when the mean firing rates are less separated, which they are when the particular cue is typical, then the probability that 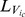 and 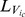 will communicate wrong instructions to 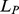 and 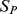 increases. This probability drives most predictions, as we shall see.

### Accumulating Evidence by Polling Neurons

As voting neurons categorize cues, polling neurons create evolving inferences about 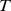. More specifically, neurons 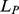 and 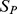 change their firing rates by increments equal to instructions received from neurons in 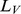 and 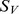, as summarized by instruction equation (2). This creates two accumulating spike rates, 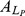 and 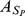, anchored at the level expected for 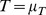, denoted 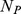, and moving up or down as instructions are received.

After all 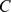 cues have been categorized, the deviation between 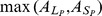 and 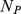 is proportional to how much the judge thinks 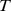 deviates from 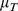. When 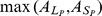  =  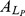 the deviation is proportional to how much the judge thinks 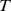 surpasses 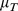, while the deviation is proportional to how much the judge thinks 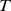 is surpassed by 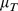 when 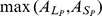  =  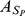.

### Converting Spikes to Relevant Scale

Since 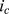 is linearly related to 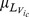 and 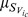, the deviation between 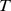 and 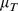 can be measured on the relevant scale by multiplying 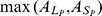 by the appropriate constant 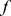. Subsequent addition of 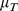 gives the judgment about 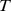:
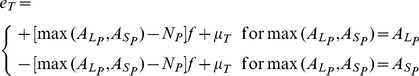
(4)


### The Truth

While judgments are probabilistic according to the model, the true value is deterministic, and so is the true deviation between 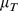 and 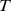. This value, henceforth referred to simply as *extremeness*, equals the sum of information values across all cues:
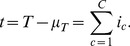
(5)


### Simplifying

**Figure 2:**
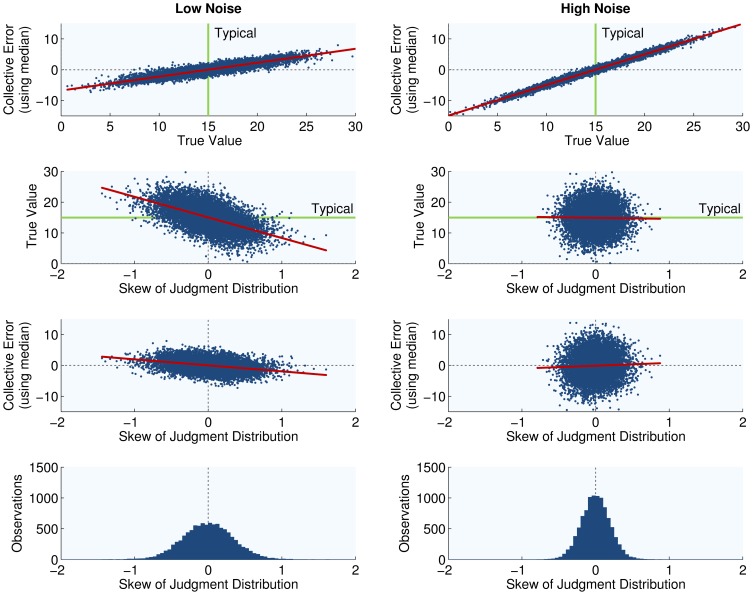
Simulation of judgment distributions using the detailed neuronal model. Two separate batches of 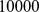 judgment distributions, formed by 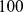 judges each, were simulated using the detailed neuronal model from which the AQ Model is distilled. Judges in the first batch were endowed with neurons categorizing information using small firing rate variance (settings of 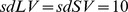 in the MATLAB code provided as Code S1, and referred to as “Low Noise”), while judges in the second batch were endowed with neurons displaying high variance (settings of 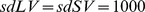, and referred to as “High Noise”). This divergence in firing rate variance produces clear differences in judgment adeptness across the batches, with judges endowed with Low Noise neurons making smaller errors on average than judges having High Noise neurons. This is because in the underlying cue categorization process that generates the intuitive judgment, the neurons of these individuals make fewer errors. Moreover, greater adeptness among judges gives rise to higher frequencies of skewed judgment distributions, and results in negative association between skew and collective error. Why that is, however, and why true value extremeness is central to these patterns, remains unclear without the AQ model.

When many competitions at the West of England Fat Stock and Poultry Exhibition are simulated (Code S1), skewed judgment distributions are often seen, and we notice negative correlation between collective error and judgment distribution skew, irrespective of us adopting the mean or the median as *vox populi* (Figure 2). Categorization error plays an important role in generating these patterns, but simplification is needed to understand how.

Things are made easier if we introduce the following assumptions: first, we restrict 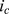 to the binary 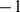 or 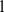; now all cues have identical absolute information values. Second, we set 
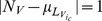
 so information, and the response to information by voting neurons, equate in absolute terms. As corollary, 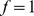 because spike rates and the true value are measured on the same scale. Third, we make 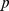 an independent and homogeneous variable across judges to understand the effect of categorization error in general. Finally, we focus on 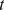 rather than 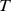, so that examined judgments concern extremeness.

**Figure 3:**
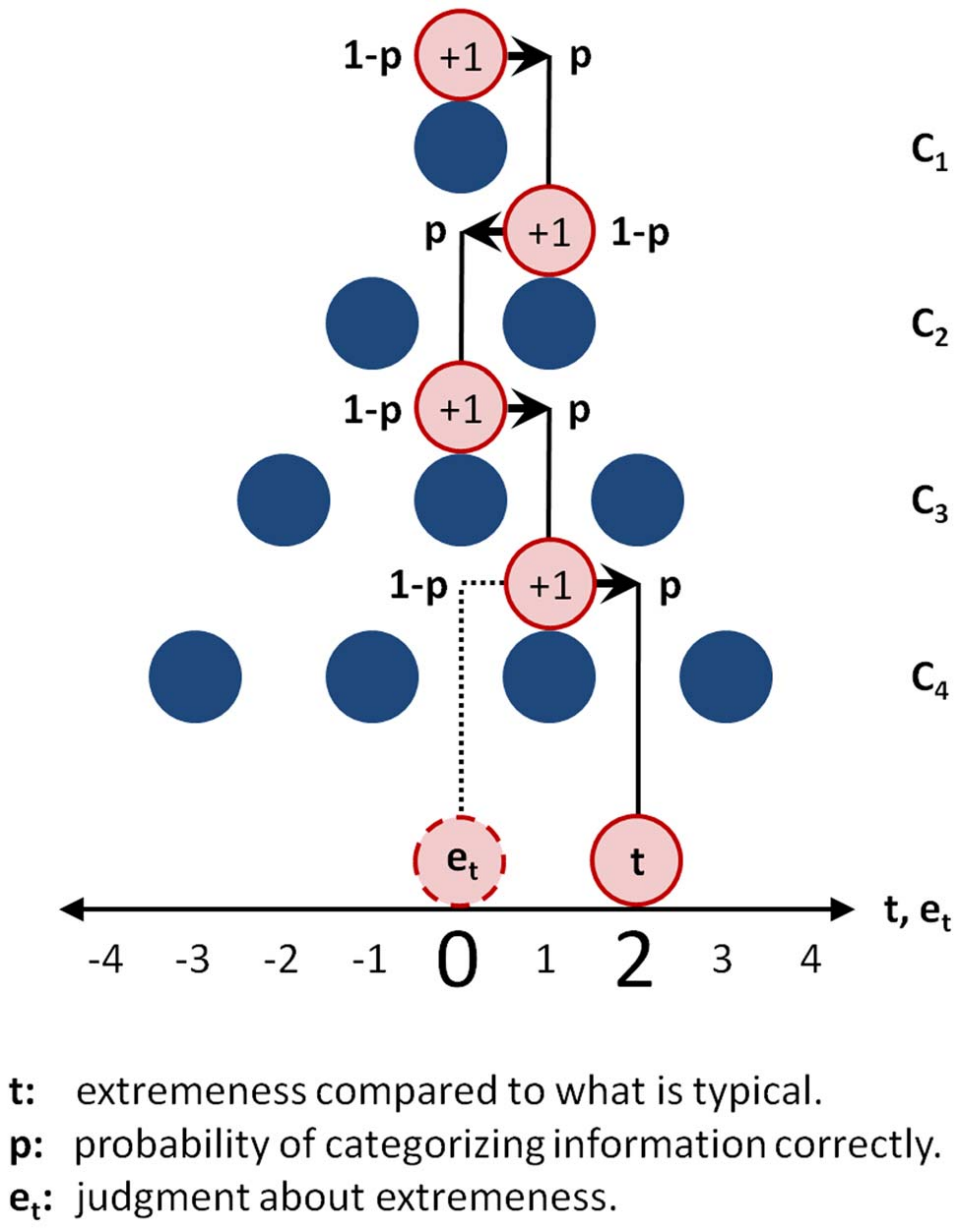
Deriving the AQ Model. The AQ model can be derived from the detailed neuronal model by assuming unit information content of cues (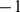 or 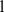), unit firing rates among voting neurons (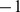 or 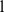), and by capturing categorization error directly (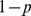). This distillation transforms the detailed model into what can be characterized as an augmented quincunx, that is to say, an augmented version of Sir Francis Galton's original probability device. Although simple, the AQ model captures the probabilistic relation between our inferences about how unusual situations are (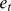) and what actually is (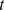), which is argued to originate from an uncertain cognitive process of categorizing information contained in cues (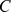). In the AQ model, rows of pegs represent cues, while balls falling through the system into one of various compartments represent the probabilistic categorization of these cues. The true value is computed by the distinct path taken by an attractor ball around pegs in the correct way.

Sir Francis Galton's [1] was the first scholar to publish ideas about how judgment distributions obtain their particular shape, but he admitted feeling uncertain about the answer due to his limited knowledge of psychology. From that perspective it is quite peculiar that distillation of the detailed model creates a *quincunx*, Sir Francis Galton's eminent probability device [11], which he invented in 1873 to demonstrate the central limit theorem. However, unlike Galton's version, which shows the dispersion of falling balls in general as they deflect past multiple rows of pins, this particular quincunx captures the probabilistic relation between the motion of balls in general, and the motion of an “attractor ball”, given probabilities of the former balls following the direction of the latter ball at every row. In this augmented quincunx model, the path of the attractor ball captures extremeness of a true value, as indicated by numerous cues (pin rows), while the other balls together reveal the probabilistic process of categorizing those cues to make an inference (the particular compartment each ball settles in) (Figure 3). I have included an application (Application S1), which the reader can use to visualize the AQ Model and its main predictions.

### Deriving the Distribution of Judgments

The distribution of judgments predicted by the AQ Model can be derived by conducting 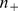 independent Bernoulli trials over cues positively associated with 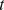, and 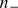 independent Bernoulli trials over cues negatively associated with 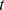. Let 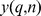 denote the number of cues out of 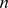 cues that are thought to be positively associated with 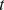 when the probability for such an opinion is 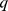.

Every judgment equals the number of cues perceived to be positively associated with 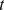, minus the number of cues perceived to be negatively associated with 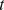, and each of these two categories can be further divided into cues perceived correctly and cues perceived incorrectly. Since these four numbers are constrained by 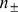 the judgment is reduced to
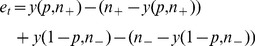
(6)

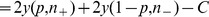
(7)


Further constraining the two independent stochastic variables 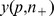 and 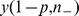, so they sum to a particular judgment, yields the distribution of judgments:
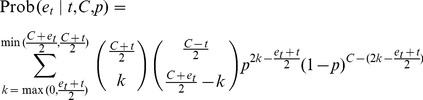
(8)


### Deriving Moments of the Judgment Distribution

While the moments of judgments can be calculated from the distribution equation (8), it is more instructive to take an alternative view of the process by which the Bernoulli trials unfold. Let the judge's categorization of one particular sequence of cues 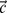 be described by 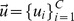, where each categorization takes on values of 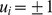, where 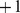 indicates correct categorization and 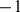 indicates incorrect categorization. Thus the sequence of categorizations represents 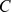 independent Bernoulli trials with the following outcomes:
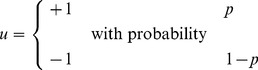
(9)


The judgment is simply the inner product of the cue and the categorization vectors:
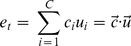
(10)


From here the moments and cross-moments of the atomic variables of equation (9) are
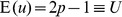
(11)

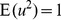
(12)

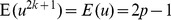
(13)

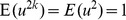
(14)

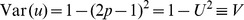
(15)

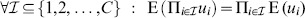
(16)

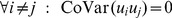
(17)and moments of the judgment distribution are calculated using these. For example, the mean judgment is derived as
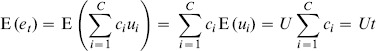
(18)


In turn, higher order raw moments can be calculated by carefully categorizing terms according to how many indices collide. For example, the second raw moment is derived as
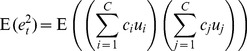
(19)

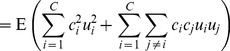
(20)

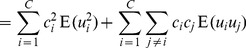
(21)

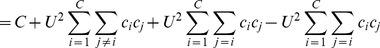
(22)

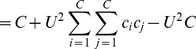
(23)

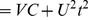
(24)noting that 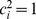. Subtracting equation (18) from equation (24), the variance 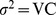 is obtained. Following this scheme, the mean, variance, and skew of the judgment distribution are found:
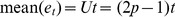
(25)

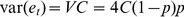
(26)

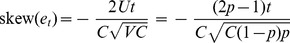
(27)


## Predictions

As described under the subheadings below, the AQ model makes four central predictions. The first prediction relates to what Galton described as the “curious anomaly” of judgment distribution skew [1], with reference to his observation in Plymouth.

### Prediction 1: Judgment Distributions are often Skewed

Setting 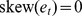 and solving for 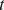, 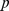, and 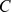 provides the conditions for judgment distribution symmetry. These conditions are 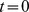 or 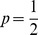, while there is no solution for 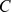. If the ox presented at Plymouth had weighed what oxen typically did in 1906, or if judges had categorized cues arbitrarily, then according to the AQ model, Galton would probably have observed a symmetric judgment distribution. However, Galton reported skew [1].

Skewed judgment distributions are predicted to occur when 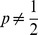 and 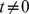, and we can hypothesize that judges at Plymouth not only confronted an ox with exceptional weight, but possessed neurons capable of categorizing the associated cues adeptly. Indeed, since the denominator of skew equation (27) is positive for all permitted values of 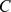, and since 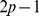 is positive for all relevant values of 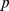, we can hypothesize judges were not just confronted with an exceptional ox broadly speaking, but were confronted with an exceptionally heavy ox. This hypothesis is made considering the negative skew reported by Galton, and noting 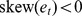 only when 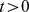. According to the AQ model, had the ox been exceptionally light, then Galton would probably have oberved positive skew.

### Prediction 2: There is Systematic Error in the Wisdom of Crowds

The AQ model predicts systematic error in the wisdom of crowds. To see this, first subtract the mean judgment equation (8) from 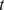 to get the expression for collective error:
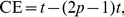
(28)


Now set 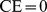 and solve for 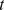 and 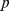. This provides the conditions where the wisdom of crowds is perfect, which it is when 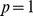 or 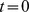. In words, the wisdom of crowds is infallible when neurons of judges make no categorization errors, or when the true value is typical, or both. Conversely, collective error is predicted when the true value is extreme and neurons categorize cues imperfectly under that condition.

The condition 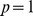 is simple to understand, because when every person makes the judgment 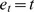, then the mean judgment equation (25) equals 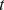, and equation (28) becomes 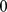. In comparison, the condition 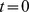 is more involved, because understanding why the wisdom of crowds can sometimes be perfect, even though neurons categorize cues arbitrarily, and why sometimes arbitrary categorization is the direct cause of collective error, can seem strange.

But consider the level of the individual and the probability of making various judgments, as captured by distribution equation (8). More specifically, consider in turn the two possible cases 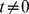 and 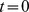. When 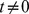 the stream of perceived information broadly agrees in the sense total evidence for the hypothesis 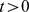 (or 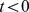) surpasses the alternative. The implication is that miscategorization of any cue supporting the correct hypothesis is more costly, because to compensate other neurons would need to miscategorize, in equal proportion, cues supporting the alternative. But these cues are rarer when 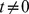. Indeed, in the most extreme case there is no tolerance for miscategorization, because there are no conflicting cues.

In contrast, when 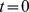 the neurons can, in principle, be exactly wrong about every cue and still cause a perfect judgment about 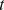, because the weight of evidence for 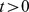 equals the weight of evidence for 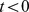. More generally, the cost of miscategorization is smallest when 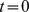 because here the probability of cancelling mistakes by chance is greatest. Furthermore, when the true value is typical, the probability is identical irrespective of the direction of miscategorization, which is untrue when the true value is extreme.

**Figure 4:**
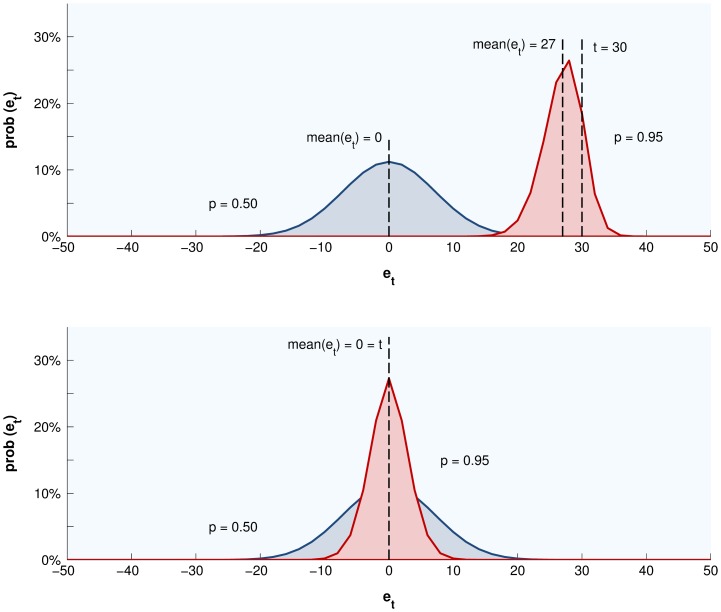
Judgment distributions formed by different crowds in typical and extreme situations. According to the AQ model, true value extremeness has no behavioral effect when crowds consists of judges whose neurons categorize cues arbitrarily (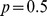). Novices cannot discriminate the true value from what is typical, and the central tendency of judgments becomes the typical value. On the other hand, adept judges (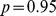 in this example) categorize information better than chance, and adjust their inference about the true value in the direction of its extremeness. However, the adjustment of individual judgments, and the adjustment of the mean, will generally be incomplete, because any miscategorization will with increasing probability move the evolving inference in the direction of the ordinary when the true value becomes more extreme. The reason is that cues pointing towards the ordinary become increasingly uncommon and therefore unlikely to be miscategorized, something required to counter the effect of miscategorizing more common cues pointing towards the extreme. On the other hand, under typical circumstances the situation is inherently fortuitous, because the true value really is typical, making the mean of judgments perfect. This is true for both novices and experts, because the probability of miscategorization is symmetric. However, the variance of expert judgment is smaller, and so are average individual errors.

Moving to the collective level again, these observations are important because they affect the probability that judgments made by many people will scatter in symmetry around the truth, and consequently the likelihood of an errorless crowd. When 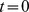 the judgment distribution tends to be symmetric for all crowds of fallible people 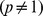, but when 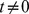 more than half the judgments tend to be smaller than 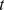 (Figure 4). Moreover, the degree to which smaller judgments outweigh larger judgments increases as 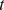 grows, and as 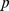 approaches 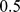. As corollary, to counter the rising effect on collective error of greater extremeness, 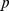 must rise, and for this reason individual adeptness is predicted to be important for collective intelligence in extreme situations, but not under typical circumstances.

### Prediction 3: The Power of Diversity is Absent

One of the most common perceptions about the wisdom of crowds is that more predictive diversity leads to greater collective intelligence. We can use the appealing *diversity prediction theorem* introduced by Page [6] to examine if the AQ model agrees. The theorem is as follows:
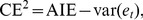
(29)where 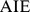 denotes average individual error. The message provided by diversity prediction theorm (8) is simple: holding average individual error constant, collective error will decrease if judgment variance is raised; apparently there are benefits to forming collectives whose members view the world as differently as possible.

But the problem with the diversity prediction theorem is equally plain. While (8) is an identity, and therefore always holds mathematically, it places no restriction on the actual relationship between 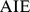 and 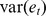, except that 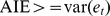. In other words, diversity might increase to compensate, or even overcompensate for greater average individual error in reality, or it might not. The diversity prediction theorem may hold either way, and cannot be used in isolation to predict if diversity actually has the effect so often bestowed upon it [6] [12]. The AQ model, however, is clear on this matter: the power of diversity is absent.

To see why, start by isolating AIE in (13) using (9) and (11) to get:
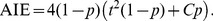
(30)


Now differentiate equation (30) with respect to 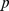, 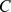 and 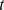 to see how 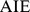 changes with these variables, and differentiate variance equation (26) with respect to 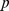 and 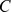 to see how 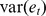 is affected; the relative movement of 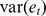 and 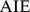 is what we must understand:
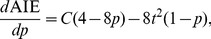
(31)

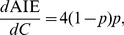
(32)

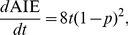
(33)

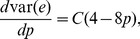
(34)and
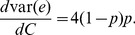
(35)


Numerous observations can be made from equation (31) to equation (35). First, notice that when 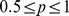, then 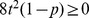 in equation (31) and 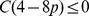 in equation (31) and equation (34). Increasing 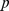 therefore never raises average individual error, nor does it ever increase the variance of judgments. Indeed, for all permitted values of 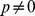, average individual error and variance both increase when 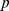 decreases, implying strictly positive association.

Second, from equation (31) and equation (34) we notice that any rise in judgment variance, produced by decreasing 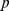, is never larger than the simultaneous rise in average individual error, implying collective error can never decrease from this effect. In other words, diversity never has positive consequence for collective error overall.

Third, the increase in diversity arising from greater numbers of cues, as indicated by equation (35), equals the simultaneous increase in average individual error indicated by equation (32), thereby cancelling the effect of raising diversity again. Finally, the rise in average individual error occurring when 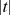 rises above 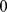 in equation (33) is not accompanied by any alleviating effect of greater diversity, because judgment variance is not predicted to change with extremeness. In short, according to the AQ model, diversity has no power to increase collective intelligence overall. On the contrary, crowds producing less predictive diversity are predicted to be wiser.

### Prediction 4: Judgment Distributions are Cues for Collective Intelligence

The AQ model predicts that when neurons categorize cues imperfectly, but not arbitrarily (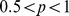), then collective error is negatively correlated with judgment distribution skew. Under this condition, and when the true value is atypically small 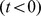, then judgment distribution skew is positive 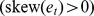 and collective error is negative 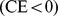, while the opposite occurs when the true value is atypically large 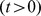.

Collective error is not predicted to cause judgment distribution skew, nor is skew predicted to cause collective error. Rather, both are caused by the presence of an extreme true value, combined with better than chance categorization of cues by neurons of judges. To see this, consider the situation where every cue points towards the same conclusion. Here perfect judgment by the crowd demands perfect categorization by every neuron of every judge, because otherwise the mean judgment will overestimate the truth when 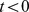, and underestimate the truth when 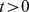.

However, while some neurons categorize accurately, others will, by chance and fallibility, categorize imperfectly, creating collective error and judgment distribution asymmetry simultaneously. As corollary, because skew is observable, it can be used to foretell collective error.

## Materials and Methods

With the aim of testing predictions of the AQ model, I gathered 3053 publicly available judgment distributions from The Federal Reserve Bank of Philadelphia's Survey of Professional Forecasters (FRBP), and The Wall Street Journal's Economic Forecasting Survey (WSJ). Distributions came from six datasets (Table 1), three from each source, and concern official measures of the US economy. From WSJ these measures were consumer price inflation (Data S1), GDP (Data S2), and unemployment (Data S3), while measures from FRBP were housing starts (Data S4), nominal GDP (NGDP) (Data S5), and unemployment (Data S6).

**Table 1:** Surveys of Expectations.

Source	Survey	Period	Frequency
The Federal Reserve Bank of Philadelphia (FRBP)	Nominal GDP	1969 to 2010	Quarterly
	Unemployment	1969 to 2010	Quarterly
	Housing Starts	1969 to 2011	Quarterly
The Wall Street Journal (WSJ)	GDP	2004 to 2011	Monthly
	Unemployment	2004 to 2011	Monthly
	Inflation	2004 to 2011	Monthly

Data was obtained from the Federal Reserve Bank of Philadelphia's Surveys of Professional Forecasters, and the Wall Street Journal's Economic Forecasting Survey. Judgments from the Federal Reserve Bank of Philadelphia about nominal GDP and housing starts concern annual percentage growth in seasonally adjusted values, while judgments about unemployment concern seasonally adjusted workforce percentages. Judgments from the Wall Street Journal also concern annual percentage growth, except for unemployment, which concerns percentages of the workforce. All judgments concern the US macroeconomic system.

Participants in each survey were economists. Surveys by FRBP are conducted quarterly, with participants required to respond midway through each focus quarter; the surveys I used were those available since initiation in 1969 until 2011. In comparison, the surveys by WSJ are conducted monthly, with participants required to respond during the first week of the focus month. Here I used available surveys since initiation in 2004 until 2011.

For the purpose of testing the AQ model, judgment data has to meet three important criteria. First, sufficient numbers of individuals must participate in each survey to provide reliable estimates of judgment distribution moments. Since 36 to 37 economists participated in each FRBP survey on average, while 54 to 55 economists participated on average in surveys from WSJ, the chosen data met this yardstick satisfactorily. Second, the forecasting methods applied by participants must involve intuition, since the AQ model is about that process. Based on information provided by Stark [13], an estimated 96 percent of FRBP economists apply intuition when forecasting, and I had no reason to suspect that participants surveyed by WSJ were different. Finally, participants must be adept judges, since the AQ model predicts that judgment distribution skew only occurs when neurons categorize cues better than chance. Since participants in all surveys are select economists, this criterion was also satisfied.

Each hypothesis was tested by calculating the Pearson correlation coefficient between variables in question, and observing levels of significance. One-sided tests were applied throughout, except for H7, where no correlation is predicted by the AQ model. Effect sizes based on Cohen's [14] classification were also reported.

### Variables

All variables except 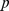 and 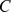 were measurable from the gathered judgment distributions, and from publicly available data on realized economic figures. The expert status of surveyed economists, however, naturally separated the range of 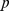 from those equal to 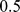, the latter being associated with judges who use information arbitrarily. In other words, I could be quite certain my analysis would say little about judgment distributions formed by novices. Moreover, the inability to measure 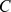 prevented me from testing the predicted effects of cue numerosity. The remaining variables, however, were operationalized as follows:

**Mean of judgments:** 
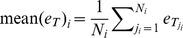
, where 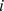 denotes the particular survey, 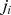 denotes the individual participant in survey 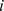, 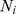 denotes the number of participants in survey 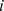, and 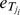 denotes the judgment submitted by participant 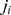.

**Variance of judgments:** 
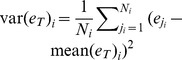
.

**Skew of judgments:** 
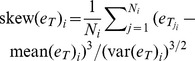
.

**Average individual error:** 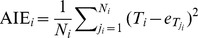, where 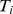 denotes the realized economic value in question for the particular survey.

**Collective error:** 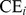  =  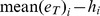. Note that 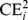 is applied in H6 below.

**Extremeness:** 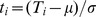, where 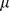 is the mean of true values across all surveys, 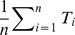, and where 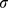 is the standard deviation of true values across these surveys, 
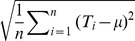
.

### Hypotheses

The status of economists as adept creates the lower bound 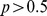, while the upper bound 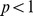 is created by the complexity of macroeconomic systems, combined with the fallibility of human judgment generally. Based on these constraints, the tested predictions of the AQ model were those listed below, with values in brackets indicating the corresponding mathematical expressions presented earlier:

**H1:** Judgment distributions have greater negative (positive) skew when prevailing true values are progressively large (small) compared to average (26).

**H2:** The mean of judgment distributions increasingly underestimates (overestimates) prevailing true values that are progressively large (small) compared to average (25)(28).

**H3:** Average individual error is greater when prevailing true values are progressively more extreme compared to average (30).

**H4:** Judgment distributions have greater negative (positive) skew when the mean of judgments underestimates (overestimates) the prevailing true value by greater margin (25)(27)(28).

**H5:** Greater judgment variance is associated with greater average individual error (31)–(34).

**H6:** Greater judgment variance is associated with greater collective error squared (31)–(34).

**H7:** There is no association between judgment distribution variance and how extreme the prevailing true value is compared to average (26).

## Results

**Figure 5:**
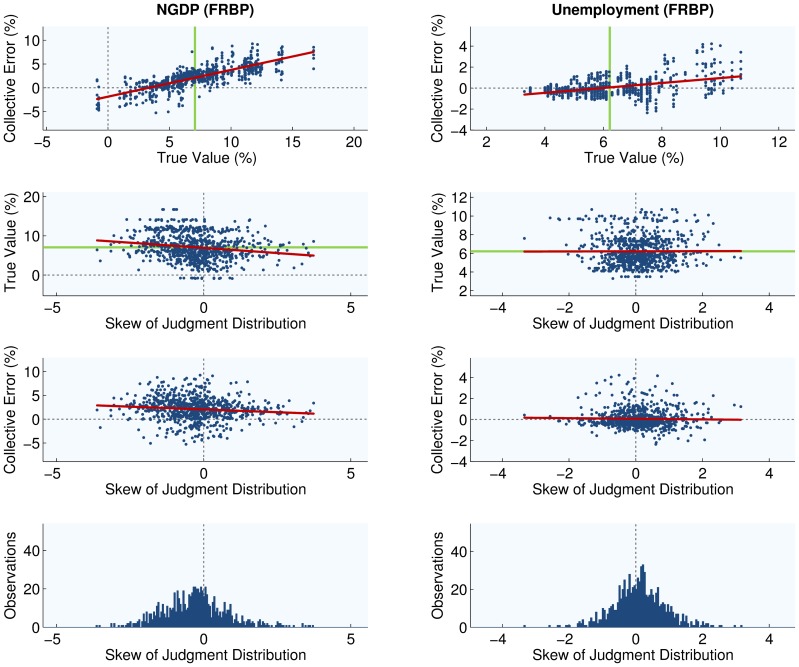
Empirical patterns of judgments about US GDP and unemployment. This figure shows associations between judgment distribution skew, collective error, and extremeness of US GDP and unemployment, compared to what is typical for these variables (solid green line). Judgments were made by economists about the annual seasonally adjusted growth of US nominal GDP, and the rate of seasonally adjusted US unemployment, as obtained from the Survey of Professional Forecasters conducted quarterly by the Federal Reserve Bank of Philadelphia.

**Figure 6:**
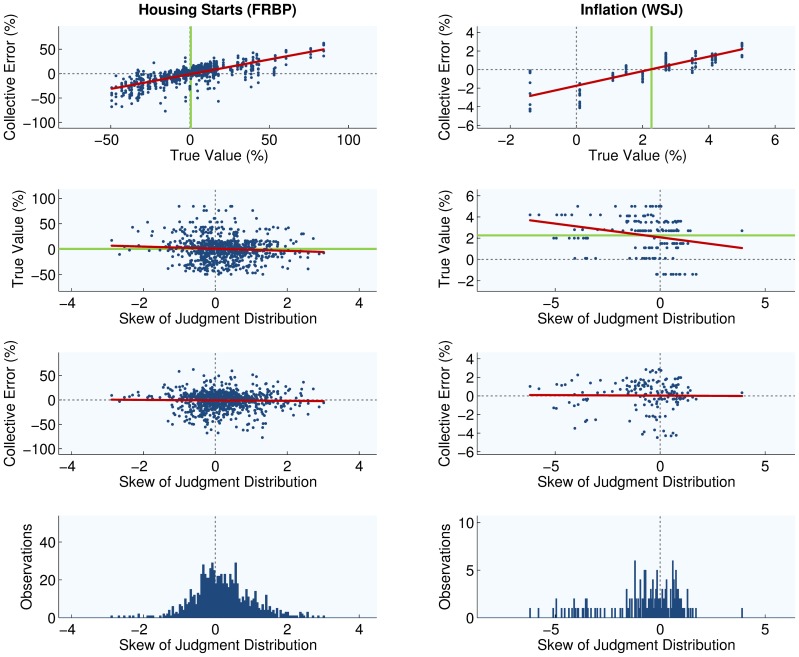
Empirical patterns of judgments about US housing starts and inflation. This figure shows associations between judgment distribution skew, collective error, and extremeness of US housing starts and consumer inflation, compared to what is typical for these variables (solid green line). Judgments in the left column were made by economists about the annual seasonally adjusted growth of US housing starts, as obtained from the Survey of Professional Forecasters conducted by the Federal Reserve Bank of Philadelphia. Judgments in the right column were made by economists about the annual rate of US consumer price inflation, as obtained from the Economic Forecasting Survey conducted monthly by the Wall Street Journal.

**Figure 7:**
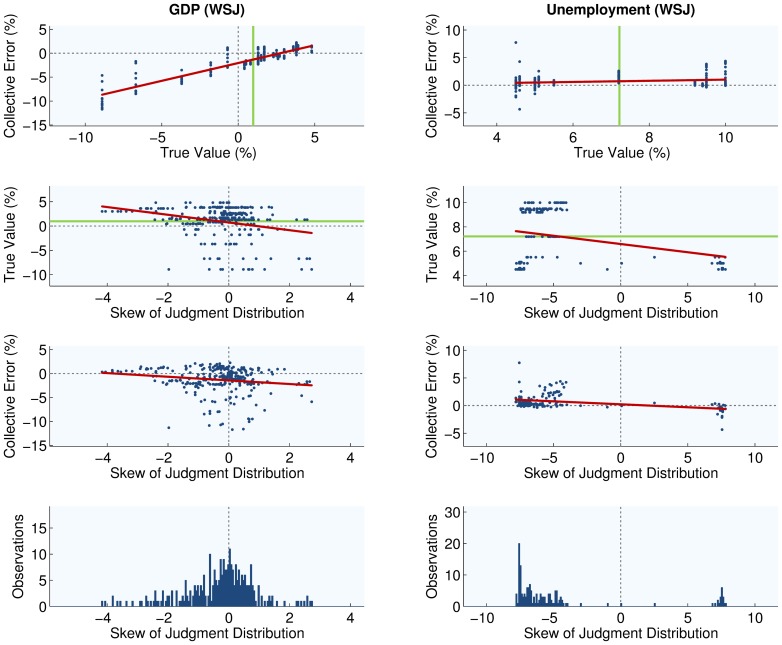
Empirical patterns of judgments about US GDP and unemployment. Shown are associations between judgment distribution skew, collective error, and extremeness of US GDP and unemployment compared to what is typical for these variables (solid green line). Judgments were made by economists about the annual growth of US GDP, and the rate of US unemployment, as obtained from the Economic Forecasting Survey conducted monthly by the Wall Street Journal.

Evidence supports the AQ model well. For visual evidence, please refer to Figure 5 for the case of judgments about US GDP and unemployment from FRBP, Figure 6 for the case of housing starts and inflation collected from FRBP and WSJ respectively, and Figure 7 for the case of judgments about US GDP and unemployment from WSJ. In 5 of 6 data sets the skew of judgments correlated negatively with extremeness (H1, Table 2). Moreover, collective error correlated positively with extremeness in all data sets (H2, Table 3), as did average individual errors in 5 of 6 cases (H3, Table 4). Hence individual economists and groups of economists generally overestimated situations where measures of the US economy were smaller than their historic levels, while they underestimated when measures were larger. Moreover, in all cases the skew of judgments correlated negatively with collective error, although signs were significant in only 3 cases (H4, Table 5).

**Table 2:** Testing H1: Negative association between 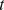 and 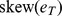.

Source	Survey	Correlation	Significance (1-tail)	Effect Size	Sign	N
FRBP	Nominal GDP	−0.180***	0.000	Small	Correct	809
	Housing Starts	−0.078*	0.013	Small	Correct	818
	Unemployment	0.004	0.453		Incorrect	819
WSJ	GDP	−0.281***	0.000	Small	Correct	287
	Inflation	−0.261***	0.000	Small	Correct	161
	Unemployment	−0.285***	0.000	Small	Correct	159

Evidence suggests that atypically large true values are associated with judgment distributions that have greater negative skew. The reverse holds for atypically small true values.

**Table 3:** Testing H2: Positive association between 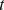 and 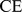.

Source	Survey	Correlation	Significance (1-tail)	Effect Size	Sign	N
FRBP	Nominal GDP	0.785***	0.000	Large	Correct	809
	Housing Starts	0.742***	0.000	Large	Correct	818
	Unemployment	0.426***	0.000	Medium	Correct	819
WSJ	GDP	0.900***	0.000	Large	Correct	287
	Inflation	0.173*	0.015	Small	Correct	161
	Unemployment	0.840***	0.000	Large	Correct	159

Evidence strongly suggests that atypically large true values are associated with positive collective error, or alternatively, that collectives underestimate atypically large true values. The reverse occurs for atypically small true values.

**Table 4:** Testing H4: Positive association between 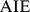 and 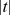.

Source	Survey	Correlation	Significance (1-tail)	Effect Size	Sign	N
FRBP	Nominal GDP	0.312***	0.000	Medium	Correct	809
	Housing Starts	0.620***	0.000	Large	Correct	818
	Unemployment	0.355***	0.000	Medium	Correct	819
WSJ	GDP	0.766***	0.000	Large	Correct	287
	Inflation	0.617**	0.001	Large	Correct	161
	Unemployment	−0.035	0.331		Incorrect	159

Evidence suggests that average individual error is greater when true values are more extreme.

**Table 5:** Testing H3: Negative association between 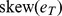 and 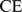.

Source	Survey	Correlation	Significance (1-tail)	Effect Size	Sign	N
FRBP	Nominal GDP	−0.111**	0.001	Small	Correct	809
	Housing Starts	−0.011	0.376		Correct	818
	Unemployment	−0.027	0.217		Correct	819
WSJ	GDP	−0.163**	0.003	Small	Correct	287
	Inflation	−0.011	0.445		Correct	161
	Unemployment	−0.372***	0.000	Medium	Correct	159

Evidence mildly suggests that negatively skewed judgment distributions are associated with positive collective error, or alternatively, that negative skew of the judgment distribution signals underestimation by the collective. The reverse occurs for positively skewed distributions.

Results were also clear on the issue of diversity. In all data sets, greater variance of judgments was positively correlated with average individual error (H5, Table 6). Moreover, when variance was greater, the associated average individual error rose in greater proportion, leading to negative correlation between collective error and judgment variance (H6, Table 7). In other words, observations of greater diversity were generally accompanied by observations of smaller collective intelligence. These findings are all consistent with the AQ model, but the final result is not: in 5 of 6 data sets the variance of judgments made by economists was found to be greater when economic measures were more extreme (H7, Table 8). In comparison, the AQ model predicts that judgment variance and extremeness are independent. For visual evidence, please refer to Figure 5, 6 and 7. Figure 5 concerns judgments about US GDP and unemployment obtained from FRBP, Figure 6 concerns judgments about US housing and inflation obtained from FRBP and WSJ respectively, while Figure 7 concerns judgments about US GDP and unemployment obtained by WSJ.

**Table 6:** Testing H5: Positive association between 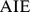 and var(e*_T_*).

Source	Survey	Correlation	Significance (1-tail)	Effect Size	Sign	N
FRBP	Nominal GDP	0.343***	0.000	Medium	Correct	809
	Housing Starts	0.482***	0.000	Medium	Correct	818
	Unemployment	0.340***	0.000	Medium	Correct	819
WSJ	GDP	0.269***	0.000	Small	Correct	287
	Ination	0.249**	0.001	Small	Correct	161
	Unemployment	0.612***	0.000	Large	Correct	159

Evidence strongly suggests that greater judgment variance is associated with greater average individual error.

**Table 7:** Testing H6: Positive association between CE^2^ and var(e*_T_*).

Source	Survey	Correlation	Significance (1-tail)	Effect Size	Sign	N
FRBP	Nominal GDP	0.304***	0.000	Medium	Correct	809
	Housing Starts	0.424***	0.000	Medium	Correct	818
	Unemployment	0.190***	0.000	Small	Correct	819
WSJ	GDP	0.193**	0.001	Small	Correct	287
	Ination	0.174*	0.014	Small	Correct	161
	Unemployment	0.804***	0.000	Large	Correct	159

Evidence strongly suggests that greater judgment variance is associated with greater collective error squared.

**Table 8:** Testing H7: No association between var(e*_T_*) and 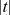.

Source	Survey	Correlation	Significance (2-tail)	Effect	Sign	N
FRBP	Nominal GDP	0.201***	0.000	Small	Inconsistent	809
	Housing Starts	0.279***	0.000	Small	Inconsistent	818
	Unemployment	0.264***	0.000	Small	Inconsistent	819
WSJ	GDP	0.392***	0.000	Medium	Inconsistent	287
	Ination	0.157*	0.046	Small	Inconsistent	161
	Unemployment	0.117	0.142		Consistent	159

Evidence suggests that judgment variance is greater when the true value is more extreme. These patterns are, unlike the patterns presented in Table 2 - Table 7, inconsistent with predictions of the AQ model under the applied assumptions. The discussion section includes an explanation of why these patterns may occur using AQ model logic.

## Discussion

I introduced and tested the augmented quincunx (AQ) model of probabilistic cue categorization by neurons of judges. My purpose was to discover if the scatter of intuitive judgments made by many different people can be predicted from the way neurons generate inferences about the environment, and to discover if the mean of judgments systematically deviates from the truth.

In the process of developing inferences about true values, when neurons categorize cues better than chance, and when the particular true value is extreme compared to what is typical and anchored upon by individual judges, then skewed judgment distributions will emerge with high probability according to the AQ model. Moreover, according to the AQ model, collective error can be inferred from the degree and direction of judgment distribution skew, and from judgment distribution variance, implying not just that judgment distributions are shaped by cues, but that judgment distributions are cues themselves for collective intelligence.

Using 3053 distributions of judgments about the US economy formed by leading economists, I found evidence supporting the AQ model. These findings suggest that trust in the wisdom of crowds should be moderated by considering the adeptness of people in the crowd, and by the particular way their judgments are observed to scatter.

### Galton's Conjecture on the Distribution of Judgments

Three important ideas are affected by this paper. The first is Galton's conjecture on the distribution of judgments, which Galton expressed in his seminal paper on the wisdom of crowds from 1907 [1]. The “curious anomaly” of the skewed judgment distributions, which Galton observed at the West of England Fat Stock and Poultry Exhibition, was thought to be caused by small varieties of different formulae among those who competed to guess the weight of an exhibited ox. The AQ model, and the presented evidence, agrees to some extent with Galton, because smaller judgment variance and greater judgment skew are predicted to occur often and together when people in the crowd are experts.

Now, we know Galton believed judges were ordinary people, but the opposing view of English botanist Perry-Coste must be considered [15]. After Galton's seminal article appeared in Nature, Perry-Coste argued with conviction to Norman Lockyer, the founding editor of Nature, that Galton had not been exposed to Vox Populi, as the title of his article indicates he believed, but Vox Expertorum. Judges were, so Perry-Coste argued, butchers and farmers whose livelihood depended on their ability to appraise the weight of farm animals before trading, and it appears to be an excellent point.

But Galton also missed another effect, namely interaction between judge adeptness and environmental extremeness; skewed judgment distributions are rare even when judges are expert, unless the subject of judgment deviates from the central tendency of prior experience.

Of course, we can only speculate about the famous ox; we know its weight, but no information was provided by Galton about its breed. All we know is that Galton described it as being “fat”. Nevertheless, my recent correspondence with Professor Van Vleck, an esteemed cattle geneticist, combined with evidence provided by McMurry [16] of Cargill Animal Nutrition, suggest judges in Plymouth were indeed presented with an exceptionally heavy specimen. According to Van Vleck, a contemporary male ox kept until maturity can reasonably weigh 2000 lb, while its dressed weight often lies in the vicinity reported by Galton. However, the situation was different back then. Due to crossbreeding, improvements in health programs, and improvements in nutrition programs, mature sizes have grown. Indeed, according to McMurry, the average bull carcass has become 30 percent heavier in the last 30 years alone. Therefore, while we cannot be sure about the particular breed, there is good reason to believe judges were presented with an exceptionally heavy ox. And this is important, because that direction of extremeness, combined with Galton's observation of negative skew and underestimation by the mean, creates circumstances exactly consistent with those the AQ model predicts.

### Muth's Rational Expectations Hypothesis

The second important idea affected by the present paper is Muth's rational expectations hypothesis from 1961 [4] [5]. Muth was right, the mean of judgments often does perform well. Nevertheless, the content of the present paper suggests the mean judgment is rational on average only, because in the particular it systematically errors depending on individual expertise and the extremeness of what is being judged.

Muth supported his assumption of rationality by noting empirical observations made by Heady and Kaldor about farmer judgments [2]. These researchers had investigated judgments about agricultural prices during 1948 and 1949, and had discovered that mean judgments across the 168 to 176 surveyed farmers corresponded to eventual prices well. What Muth did not disclose, however, but what Heady and Kaldor had made clear, was that 3 of 4 examined judgment distributions were noticeably skewed, while the last distribution was “nearly normal”. More precisely, all distributions were more or less positively skewed, and their mean overestimated actual prices in 3 of 4 cases by 8 to 27 percent, while the actual price was underestimated by 1 percent in the final case. Given the present paper we recognize these observations as consistent with predictions of systematic error in the wisdom of crowds, and thereby systematic violation of Muth's influential assumption about collective intelligence.

### Page's Diversity Prediction Theorem

The final idea affected by the content of the present paper is the power of diversity to reduce collective error, as argued by Page using his diversity prediction theorem [6]. While transition from individual, to group, and finally to crowd, will involve the introduction of predictive diversity whenever people are fallible, and while such diversity will introduce collective intelligence, it should not be concluded in haste that introducing greater diversity to already established crowds is beneficial too. Indeed, from the practical perspective of assembling collectives, the designer must be careful to separate the beneficial effects of increasing the number of collective members, from the costly effects of introducing individuals to crowds who are less adept than average.

### Weaknesses and Strengths

At this point an apparent weakness must be stated, which limits the scope of supported conclusions about diversity. I wrote the above to coincide with the predictions and findings presented, but the logic of the AQ model leads to the idea that diversity can, contrary to predictions under the chosen assumptions, be positively associated with 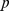, and that increasing the diversity of crowds can be beneficial sometimes after all. I have throughout assumed homogeneity in the probability of categorization error, but let us consider a situation where homogeneous novices are joined by experts, creating an assortment of 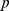 in the collective. Furthermore, let us assume this event happens under extreme circumstances.

Before the experts arrived, collective error would be quite substantial (revisit Figure 3), but two things now happen. First, the mean judgment moves farther away from the typical value and closer towards the truth, driven by smaller individual errors among the newcomers. Second, the variance of judgments increases. In other words, contrary to predictions under the assumption of homogeneity, there is reason to suspect introducing more diversity can be positive, if the increase in diversity is caused by adept newcomers, and if the situation is extreme.

Yet the assumption of homogeneity appears to generate another weakness too, namely the discovered inability to explain why predictive diversity correlates positively with extremeness (revisit Table 8). To see this, let us continue our story and let us assume the new collection of heterogeneous judges must now evaluate numerous true values in succession. Under typical circumstances the distribution of judgments will centre on the truth, with variance determined mainly by errors among the incumbents. But as the true value becomes more extreme, the combined behaviour of experts and novices becomes pivotal. Judges whose neurons categorize cues arbitrarily will be unresponsive to how extreme the true value is, while adept judges will form judgments moving with the truth. In other words, for the entire collective of heterogeneous judges the spread of judgments increases with extremeness, and the inconsistencies shown in Table 8 are thereby explained. In passing, note that skew is amplified during this process by the combination of unresponsive novices, and experts tuned to developments. Indeed, using the application supplied in Application S1, the reader can verify these claims.

Meanwhile, other assumptions can also be debated. First, there is the assumption individuals form judgments by using the central tendency of prior experience as their initial thought, and adjust away from this anchor as unusual information is received. An alternative approach would be to assume individuals anchor at zero, and adjust their inference up or down by the full information value of categorized cues, as is common when judgments are modeled using regression [17] [18]. Either way, reference point logic is applied in ways consistent with neurons accumulating discrete evidence sequentially and probabilistically, but only the chosen approach is consistent with established ideas on self-generated anchoring [19]. Indeed, the chosen assumption leads to conclusions relevant for research on the anchoring heuristic, by providing an explanation for the phenomenon of incomplete adjustment [20].

Second, the assumption individuals have generated identical anchors appears unrealistic for novices with little experience, but for individuals with substantial experience working on the same problem, the assumption appears unproblematic. Indeed, substantial reference point diversity among experts would need explaining.

Third, in reality many aspects of the environment contain redundant information, because they are not independent of other aspects. I have assumed stochastically independent cues, which may, from that perspective, be considered wrong. However, while many aspects of the environment correlate, most correlate imperfectly, which implies that despite carrying some redundant information, not all information carried by dependent aspects is superfluous. Indeed, the assumption of stochastically independent cues can be viewed as an assumption of modularity in the environment, with cues being defined as these modules. Within modules there is correlation between different elements, while between modules little correlation exists.

Fourth, the discrete nature of the modeled probabilistic process leads to discrete judgment distributions unless neurons categorize many cues, while more continuous distributions usually arise in reality, even when judges process little information. This, however, is an inconsistency diminished simply by introducing exogenous noise, as is common when judgments are modeled using regression. Alternatively, the assumption that only two competing voting neurons are activated by the particular cue can be eased to create *tuning curves* [21]–[25]. Now polling neurons adjacent to the one with greatest mean response might by chance demonstrate greatest activity, thereby decoupling information value from encoded evidence in random fashion. Whatever approach is taken, however, predictions will be unaffected unless noise removes all neural correlates of information.

Finally, the introduced model appears confined to non-social processes, since the judgments made by different individuals are assumed to be independent draws from the same probabilistic process. Nevertheless, this observation is only true in part, because no assumptions are made about cues being communicated socially or not. What is not captured, however, is the display of misleading cues, or the use of social influence to affect how others categorize cues. Moreover, any effect that limits access to cues, such as social networks, is not captured either.

Nonetheless, despite forces undoubtedly being unrepresented in my argument, the AQ model does an excellent job predicting and explaining the phenomenon it was constructed to help us understand. Moreover, it does so, after all, with higher levels of realism than common least square models of intuitive judgment [29]. Indeed, from the perspective of cue learning psychology, the introduced model is consistent with Brunswik's [26] [27] [28] ideas on probabilistic functionalism, and predicts that Brunswik's most basic subject of study, namely the adjustment of the organism to the environment, is observable from the shape of judgment distributions formed by many facing this problem of cognitive adaptation.

In short, the results I have presented link neuroscience to the social realm in ways consistent with cue learning psychology. As people struggle to adjust to their environment, neurons in the brain encode information mediated by the environment to generate phenomena observable not just from the individual, that is to say, from the intuitive judgments people make, but from the crowd, that is to say, from the distribution of judgments made by many different people. That discovery is significant because it moves scientists across these fields closer to understanding the success and failure of information processing entities at multiple levels of aggregation, from individual neurons to entire human organizations.

### Future Research

Future research on systematic error in the wisdom of crowds will likely be done using massive data sets of judgments, using controlled laboratory settings, or ideally, using controlled settings generating massive data. In the immediate future it remains to be examined if cue numerosity really does increase both diversity and average individual error, and if skewed judgment distributions are formed less frequently by novices as predicted. These examinations hold the promise of improving our confidence in the specific probabilistic representation depicted by the AQ model. Such confidence would be particularly boosted, however, if predictions about skew, collective error, and extremeness, were also demonstrated to hold for *the crowd within* [30] [31] [32]. At that point what promises to be the most rewarding continuation could then be initiated, namely cue learning experiments among crowds of participants, where algorithms extract relationships between collective error and judgment distribution skew in real-time, for the purpose of correcting mistakes before they occur in said laboratories, and beyond.

Meanwhile, however, the robustness of candidate procedures can already be tested through bootstrapping on existing data sets of substantial size. Indeed, the most obvious candidate process involves merely three parameters: 1) the number of judgment distributions used to linearly estimate the association between collective error and skew, 2) the number of judges used to form these distributions, and 3) the number of judges used to generate the cue for collective error on the next task. Within the constraint of each particular setting, random batches of judges and tasks would be sampled to discover the average error of the skew-adjusted mean, and the variance of this error, so comparisons can be made with errors generated by the wisdom of the crowds, as measured by the arithmetic mean, or the median of judgments.

## Conclusion

Every day is filled with decisions based on intuitive judgments. When doctors diagnose patients, their intuitive judgments affect the choice of treatment and the patient's subsequent chance of recovery. When managers assess company performance, their intuitive judgments affect jobs and the economic livelihood of workers. And when individuals vote for politicians, their decision is based on intuitive judgments about how well the politician will perform in office, with the prosperity of entire nations sometimes at stake. We have long known that harnessing the wisdom of crowds can help us reduce the extent of our individual errors, yet what the present paper indicates is that our trust in popular judgment should have limits, because there is systematic error in our collective intelligence, emerging from the way our neurons categorize information contained in the cues we use. But as with all previous discoveries of judgment biases, now we know, we are positioned to do something about it.

## Supporting Information

Application S1 **Zipped Java application that readers can use to become familiar with the AQ Model, and discover what predictions it makes about judgment distributions and collective error.** The application is accompanied by further instructions.
(ZIP)

Code S1 **Zipped MATLAB code for simulating many judgment competitions of the kind observed by Sir Francis Galton in 1906.** Judgments made by each competitor derive from the modeled behaviour of their neurons. Readers may toggle between Gaussian or Poisson distributed neuronal firing, and may also examine the effect of firing rate variance on the character of the judgment distribution that judges collectively form.
(ZIP)

Data S1 **Judgment data in Excel about US consumer price inflation, gathered from The Wall Street Journal's Economic Forecasting Survey.** Judgments concern annual percentage growth. Surveys are conducted monthly by the journal.
(XLS)

Data S2 **Judgment data in Excel about US GDP, gathered from The Wall Street Journal's Economic Forecasting Survey.** Judgments concern annual percentage growth. Surveys are conducted monthly by the journal.
(XLS)

Data S3 **Judgment data in Excel about US Unemployment, gathered from The Wall Street Journal's Economic Forecasting Survey.** Judgments concern percentage of the workforce. Surveys are conducted monthly by the journal.
(XLS)

Data S4 **Judgment data in Excel about US housing starts, gathered from The Federal Reserve Bank of Philadelphia's Survey of Professional Forecasters.** Judgments concern annual percentage growth in seasonally adjusted values. Surveys are conducted quarterly by the bank.
(XLS)

Data S5 **Judgment data in Excel about US nominal GDP, gathered from The Federal Reserve Bank of Philadelphia's Survey of Professional Forecasters.** Judgments concern annual percentage growth in seasonally adjusted values. Surveys are conducted quarterly by the bank.
(XLS)

Data S6 **Judgment data in Excel about US unemployment, gathered from The Federal Reserve Bank of Philadelphia's Survey of Professional Forecasters.** Judgments concern seasonally adjusted percentages of the workforce. Surveys are conducted quarterly by the bank.
(XLS)
